# Active search for antecedents in cataphoric pronoun resolution

**DOI:** 10.3389/fpsyg.2015.01638

**Published:** 2015-10-30

**Authors:** Leticia Pablos, Jenny Doetjes, Bobby Ruijgrok, Lisa L.-S. Cheng

**Affiliations:** ^1^Leiden University Center for Linguistics, Leiden UniversityLeiden, Netherlands; ^2^Leiden Institute for Brain and Cognition, Leiden UniversityLeiden, Netherlands

**Keywords:** cataphora, active search, gender mismatch, anterior negativity, Principle C

## Abstract

Cataphoric dependencies where a pronoun precedes its antecedent appear to call on different mechanisms in language comprehension from forward dependencies where the antecedent precedes the pronoun. Previous research has shown that the resolution of cataphoric dependencies involves predictive processes such as the active search mechanism, which hypothesizes the automatic search for an antecedent immediately after encountering a cataphoric pronoun. The current study employs gender mismatch to investigate whether the active search for an antecedent of a cataphoric pronoun is restricted only to grammatically licit positions. We present results from an event-related potential experiment on the reading comprehension of cataphoric dependencies in Dutch. Results show that gender mismatch gives rise to an anterior negativity at grammatically licit antecedent positions only. We hypothesize that this negativity reflects the prediction failure for an antecedent after encountering a pronoun, rather than a gender mismatch. We discuss the timing, topography and functionality of this negativity with respect to previous studies and how this relates to the ERPs elicited in the processing of structural constraints on pronoun resolution.

## Introduction

The on-line interpretation of pronominal dependencies has raised several questions within theories of sentence comprehension. Forward pronominal dependencies – where the antecedent precedes the pronoun – and backward pronominal dependencies – where the pronoun precedes the antecedent – appear to call on different mechanisms in language comprehension. In the case of forward dependencies, their resolution requires retrieving the information about the antecedent at the position of the pronoun, which is closely connected with memory-retrieval processes (Chow et al., 2014). On the other hand, the resolution of backward dependencies (also called cataphoric dependencies) requires the search for an antecedent, which is related to predictive processes.

One of such predictive processes is the active search mechanism (ASM), found initially for the interpretation of *wh*-gap dependencies (Crain and Fodor, 1985; Stowe, 1986; Clifton and Frazier, 1989). In the case of backward dependencies, the ASM hypothesizes that the human parser automatically starts a search for an antecedent in the upcoming sentence immediately after encountering a cataphoric pronoun. This has been shown in behavioral studies through gender mismatch effect (GMME) observations in experimental paradigms where possible antecedents for cataphoric pronouns are restricted by grammatical principles (Sturt, 2003; Van Gompel and Liversedge, 2003; Kazanina et al., 2007; Yoshida et al., 2014). This paper presents an event related potential (ERP) study where we confirm that a similar effect can also be observed in neurophysiological data. Results support the presence of an ASM for cataphoric dependency resolution that respects grammatical principles. The topography and timing of the ERP component generated at the mismatching antecedent position in our study was an anterior negativity, while previous forward antecedent/pronoun dependencies studies have found a P600 (Osterhout and Mobley, 1995; Van Berkum et al., 2007; Xu et al., 2013). We postulate that the ERP component observed in cataphoric dependencies is related to a failure of a prediction by the parser, in line with the active search approach, while in the case of forward antecedent dependencies the effect can only be connected to a gender mismatch as no prediction is made (the pronoun is not required to interpret the antecedent).

### Cataphoric Dependencies

Cataphoric pronouns are pronouns that occur linearly before their antecedent. In other words, they are instances of referential dependencies in which the antecedent follows the referentially dependent element, as illustrated in (1). The index *i* indicates that *he_i_* and *Peter_i_* refer to the same person.

(1) While **he_**i**_** had a broken arm, **Peter_**i**_** could not ride his bike.

Pronouns such as *he* in (1) pose an interesting case for parsing theories. In order to resolve the interpretation of the pronoun with an antecedent in the same sentence, the parser needs to wait until the appearance of the antecedent. When the antecedent is found, the pronoun can establish a link with it for its own interpretation. However, this is only possible when the grammar allows the link between the cataphoric pronoun and the antecedent to be established. Consider the pronoun *he* in (2) and the pronoun *his* in (3). In contrast to the pronoun in (1), the pronoun in (2) cannot take the proper name *Peter* as its antecedent (as indicated by the starred index of *j* – *he* cannot have the same index/reference as *Peter*). However, *Peter* can be the antecedent of the pronoun *his* in (3).

(2) **He**_**i**/∗j_ said that **Peter**_**j**_ is sick.
(3) **His_**i**_** brother said that **Peter_**i**_** is sick.

The restriction of the pronominal reference in (1), (2), and (3) can be captured under the principles of the Binding Theory (Chomsky, 1981) that indicates the configurations in which nominal elements can or cannot establish a coreferential relation. There are three Binding Principles, each of which concerns a different type of nominal elements. Binding Principles A and B are concerned with two different types of pronouns (*himself* vs. *him*), while Principle C restricts the distribution of Referential Expressions, including proper names such as *Peter*.

We focus on Principle C, which prohibits a Referential Expression (e.g., proper name) from being *bound* (Chomsky, 1981). The pronoun *he* in (1) does not bind the referential expression *Peter*, because the pronoun is embedded in an adverbial clause that does not contain *Peter*. Given that *he* does not bind *Peter*, the two can have the same reference. On the other hand, the pronoun *he* in (2) binds the referential expression *Peter* structurally and in such a case, coreference is excluded. *His* in (3) on the other hand is more deeply embedded in the structure (i.e., in the noun phrase *his brother*), and therefore, it does not act as a binder of *Peter*. Thus, similar to (1), a cataphoric dependency can be established in (3). Referential expressions, such as *John* or *the man*, independently refer and select a referent from the domain of discourse. Given that Referential Expressions have independent reference, they do not need and in fact cannot tolerate a binder. The binder would act as an antecedent for the Referential Expression, which is in conflict with the referential status of the latter.

In this study, we investigate whether Principle C of the Binding Theory is respected in cataphoric pronoun processing. As illustrated in (1), (2), and (3), whether a referential expression can be a potential antecedent for a cataphoric pronoun depends on the structural configuration. If a coreferential relation is established between a referential expression and a cataphoric pronoun and as a result, the referential expression is bound by the pronoun, Principle C of the Binding Theory would be violated. This paper examines how this type of violation affects parsing. In particular, it uses gender mismatch to investigate whether a search for an antecedent is restricted by structural constraints. Given that the parser respects structural constraints such as Principles B and C of the Binding Theory when interpreting pronouns on-line as shown by behavioral studies that have examined reading times (e.g., Kazanina et al., 2007; Chow et al., 2014; Yoshida et al., 2014), we expect these effects to be visible through electroencephalography (EEG) as well.

### Active Search Mechanism [or Active Filler Hypothesis (AFH)]

The ASM claims that an active search is automatically initiated for each uninterpreted element A encountered in a sentence, to find the element B which can help interpret A. The main evidence for the existence of the active search comes from the so-called filled-gap effects involving *wh*-dependencies, which demonstrate that (a) a search for a gap starts as soon as a *wh*-phrase is processed and (b) filling the gap position where the *wh*-word could be interpreted with an overt element (thus blocking the parser from interpreting the *wh*-phrase in that position) results in a longer processing time compared to a sentence where no *wh*-dependency was initiated (Crain and Fodor, 1985; Stowe, 1986; Lee, 2004). Thus, the ASM hypothesizes that the parser anticipates a gap as soon as a *wh*-phrase is processed (Clifton and Frazier, 1989; Frazier and Clifton, 1989).

In the case of pronoun interpretation, the ASM predicts that a search is initiated for an antecedent as soon as a pronoun is encountered (Clifton and Frazier, 1989; Kazanina et al., 2007), in order to resolve the interpretation of the pronoun. Even though pronouns may have antecedents outside of the sentence that contains them, the ASM assumes that the search for an antecedent within the sentence is the default strategy in cases where there is no preceding discourse.

Studies on the processing of cataphoric pronouns have examined whether the parser indeed searches for an antecedent in the sentence once a pronoun has been processed and when the grammar allows the establishment of the binding relation (Sturt, 2003; Van Gompel and Liversedge, 2003; Kazanina et al., 2007; Yoshida et al., 2014). In these behavioral studies, which used eye-tracking or self-paced reading methodology, the parser searches for an antecedent in the upcoming input in positions where the coreference between the pronoun and the antecedent is allowed (i.e., such coreference does not lead to a violation of the Binding Theory). In such cases, when the potential antecedent does not match in gender with the preceding pronoun, reading times are longer than when the potential antecedent and the pronoun match in gender (Sturt, 2003; Van Gompel and Liversedge, 2003; Kazanina et al., 2007; Yoshida et al., 2014). This reading slowdown effect, known as the GMME, has been taken to be a sign of the parser’s active search for an antecedent to interpret the pronoun. Importantly, the data in these studies show that the GMME does not occur if the coreference between the pronoun and the referential expression yields a violation of the Binding Theory (in particular, Condition C), suggesting that in such cases, the referential expression does not count as a potential antecedent for the pronoun.

The main hypothesis of Kazanina et al. (2007) word-by-word self-paced reading experiments is that the parser respects Principle C of the Binding Theory when searching for an appropriate antecedent for a pronoun. This can be illustrated on the basis of the four different conditions in (4), which are from their third experiment: no constraint match in (4a), no constraint mismatch in (4b), Principle C match in (4c) and Principle C mismatch in (4d).

(4) a. **No constraint/Match**
    **His_i_** managers chatted amiably with some fans while **the talented, young quarterback_**i**_** signed autographs for the kids, but **Carol** wished the children’s charity event would end soon so she could go home.
    **b. No constraint/Mismatch**
    **Her_i_** managers chatted amiably with some fans while **the talented, young quarterback** signed autographs for the kids, but **Carol_**i**_** wished the children’s charity event would end soon so she could go home.
    **c. Principle C/Match**
    **He_**i**_** chatted amiably with some fans while **the talented, young quarterback** signed autographs for the kids, but **Steve_**i**_** wished the children’s charity event would end soon so he could go home.
    **d. Principle C/Mismatch**
    **She_**i**_** chatted amiably with some fans while **the talented, young quarterback** signed autographs for the kids, but **Carol_**i**_** wished the children’s charity event would end soon so she could go home.

In the no constraint match condition in (4a), the possessive pronoun *his*, being further embedded in the nominal structure, does not bind the referential expression *young quarterback*, allowing it to be a potential antecedent. In other words, in (4a), Principle C does not block the coreference relation between the pronoun *his* and the referential expression (the antecedent *young quarterback*), and these two elements match in gender. Therefore the cataphoric pronoun should be interpreted at the antecedent position. The no constraint mismatch condition in (4b) differs from the no constraint match condition in (4a), in that the gender of the pronoun *her* and that of the potential antecedent *young quarterback* do not match, creating a GMME. In the Principle C match condition in (4c), on the other hand, the pronoun *he* binds the referential expression *young quarterback* in the embedded clause. Thus, *young quarterback* is excluded as a potential antecedent of *he* due to a Principle C violation. Furthermore, both the pronoun *he* and the referential expression *young quarterback* match in gender, as both are masculine. Finally, in the Principle C mismatch condition in (4d) the pronoun *she*, binds the referential expression *young quarterback* in the embedded clause, just like in (4c); however, in this case, they mismatch in gender. Importantly, the GMME is expected to be absent in the Principle C mismatch condition (condition 4d) at the position of the referential expression *young quarterback*, relative to the Principle C match condition (4c), as the coreference relation is barred from being established due to Principle C, preventing the GMME to occur. Conversely, the GMME is expected to be present at the referential expression *young quarterback* position in the no constraint mismatch condition (4b), relative to the no constraint match condition (4a). The main findings of Kazanina et al. (2007) confirm these expectations. Their reading time results thus suggest that the parser abides by Principle C when it attempts to resolve the interpretation of cataphoric pronouns in real-time in that they only find a reading time difference, or GMME, in the no constraint conditions, in which the referential expression in the no constraint mismatch condition in (4b) elicited longer reading times than the no constraint match condition (4a) at the same position (in particular, at the noun *quarterback*), whereas this reading time difference was absent at the referential expression in the Principle C conditions in (4c) and (4d). Furthermore, Kazanina et al. (2007) claim that the active search for an antecedent in cataphoric configurations only occurs when the Binding Principles allow it.

Yoshida et al. (2014) examine the formation of cataphoric dependencies across a relative clause island in a word-by-word self-paced reading experiment and they expect to obtain a GMME, or longer reading times, only in cases where coreference between the pronoun and the antecedent is licit (i.e., not obeying Principle C). Further, the GMME would only be expected to occur if cataphoric dependencies were not to be sanctioned across relative clause islands. Similar to Kazanina et al. (2007), Yoshida et al. (2014) manipulated the sentence initial pronoun [nominative vs. (possessive) genitive], the gender of the pronoun and the first referential expression. Their stimuli are shown in (5). In (5a) and (5b) the pronouns *his/her* can corefer with the referential expression *Jeffrey Stewart* (thus, *Jeffrey Stewart* can be a potential antecedent), but in (5c) and (5d) coreference is not licit due to Principle C of the Binding Theory.

(5) **a. No Constraint/Match**
    **His_**i**_** managers revealed that the studio that notified **Jeffrey Stewart**_**i**_ about the new film selected a novel for the script, but **Annie** did not seem to be interested in this information.
    **b. No constraint/Mismatch**
    **Her_**i**_** managers revealed that the studio that notified **Jeffrey Stewart** about the new film selected a novel for the script, but **Annie**_**i**_ did not seem to be interested in this information.
    **c. Principle C/Match**
    **He_**i**_** revealed that the studio that notified **Jeffrey Stewart** about the new film selected a novel for the script, but **Andy**_**i**_ did not know which one.
    **d. Principle C/Mismatch**
    **She_**i**_** revealed that the studio that notified **Jeffrey Stewart** about the new film selected a novel for the script, but **Annie_**i**_** did not know which one.

A GMME or reading slowdown is found at the antecedent position *Jeffrey Stewart* (in particular, at the last name *Stewart*) in (5b) relative to (5a), where the pronoun and the antecedent could corefer (the coreference does not violate Principle C). Moreover, the GMME or reading time difference occurs despite the fact that the potential antecedent is contained within a relative clause island. The GMME generated in the no constraint conditions (5a) and (5b) in the self-paced reading experiment by Yoshida et al. (2014) confirms that online formation of a cataphoric dependency is not affected by island constraints in that coreference is established in (5a) and (5b) conditions when the grammatical constraint of Principle C does not ban this coreference. If island constraints affected the generation of a cataphoric dependency we will not expect a GMME to occur in no constraint conditions, which it does. Furthermore, these results support the claim in Kazanina et al. (2007), that the processing of cataphoric dependencies is modulated by a grammatically constrained ASM, which respects grammatical principles such as Principle C.

The current study aims to replicate the GMME results from previous studies (Sturt, 2003; Van Gompel and Liversedge, 2003; Kazanina et al., 2007; Yoshida et al., 2014; a.o.) using ERP, to identify a neural correlate of the ASM found in the on-line interpretation of cataphoric dependencies. If an active-search is initiated for these dependencies (as shown by previous behavioral studies through the generation of the GMME effect, which is a slowdown in the gender mismatching conditions relative to the gender matched ones), it should be possible to identify an effect (i.e., an ERP component) comparable to the reading time differences shown in behavioral studies with the ERP methodology. In other words, we predict there to be a GMME in the no constraint mismatch conditions such as (4b) and (5b) above, relative to the no constraint match conditions in (4a) and (5a).

### Event-related Potential (ERP) Studies on Gender Agreement/Mismatch

Since the current study examines gender agreement mismatches at the antecedent position in cataphoric configurations, a brief overview of the ERP studies that have tackled gender agreement issues is in order. Gender agreement mismatches have been examined in the ERP literature using different paradigms. Wicha et al. (2004) found a P600 for gender disagreeing nouns in determiner-noun combinations in Spanish, where the expected noun mismatched in gender with the preceding determiner. Van Berkum et al. (2005) on the other hand tested the prediction for the likely appearance of a specific noun based on the previous discourse. Their aim was to examine how listeners use their discourse knowledge to predict specific nouns. If listeners anticipate a noun with a specific gender by the time they encounter the indefinite article (not gender marked) in the story, a gender-mismatched adjective (i.e., mismatched in accordance to the gender of the noun that is expected) would be a surprise, leading to an ERP effect at the adjective position. They tested Dutch sentences where the sentence continuations had either an adjective consistently gender-marked with the upcoming predicted noun and its gender, or an adjective inconsistently gender-marked with respect to the prediction made for the upcoming noun and its gender. Their results again showed a P600 for gender-mismatched adjectives.

In a different set of studies, gender agreement violations between a determiner and a noun, or between an adjective and a noun, showed a left anterior negativity (LAN) followed by a P600 at the noun position for Spanish, Italian, and German (Demestre et al., 1999; Gunter et al., 2000; Barber and Carreiras, 2005; Molinaro et al., 2008; a.o.), a P600 for English and Dutch (Hagoort and Brown, 1999) and a N400 followed by a P600 for Hebrew (Deutsch and Bentin, 2001).

Finally, in a third set of studies, gender violations were tested in forward pronoun resolution dependencies, i.e., dependencies in which antecedents occur before pronouns. Osterhout and Mobley (1995) tested sentences such as (6) where a masculine or feminine pronoun matched or mismatched in gender with a previously encountered antecedent. They found a P600 at the pronoun *he* that mismatched in gender with the previously encountered feminine antecedent *the aunt*. Note that coreference between *he* and *the aunt* is only blocked by the gender mismatch and not by the Binding Conditions, as pronouns, contrary to referential expressions, may be bound by their antecedent if the antecedent is located in a different clause (cf. Principle B of the binding theory).

(6) **The aunt** heard that **she/he** had won the lottery.

Similarly, studies that tested gender violations in comparable forward pronoun configurations in Dutch (Van Berkum et al., 2007) and Chinese (Xu et al., 2013) found a P600 at the position of the pronoun when it mismatched in gender with the preceding antecedent.

Taking into consideration the results in these studies that have manipulated gender agreement, it is clear that a P600 component emerges constantly, regardless of whether the relation is one between (1) a determiner and a noun; (2) an adjective and a noun; or (3) an antecedent and a pronoun. While the P600 is preceded by a LAN or by a N400 in some cases in pure pronoun resolution cases more akin to the manipulation in the current study, only a P600 is obtained at the position of the gender-mismatched pronoun.

### The Current Study

As indicated above, the present study examines processing of pronouns and their antecedents in a *cataphoric configuration*, where the pronoun linearly precedes the antecedent. To summarize, the aim of this study is threefold. (i) First is to examine whether there is a GMME when the parser encounters the first potential antecedent of the cataphoric pronoun that does not match in gender. This would be an indication that the parser starts actively searching for a matching antecedent after encountering the cataphoric pronoun, even though the antecedent of the pronoun could, in principle, be found outside of the sentence. We predict the GMME to be present in the case of a mismatch, and absent in the matching condition. (ii) Second, we examine if the search mechanism is modulated by grammatical constraints such as Principle C of the Binding Theory. For cases where co-reference may lead to Principle C violations, we predict no difference between the match and the mismatch conditions. We predict that an ERP component is elicited only for referential expressions that can legitimately establish a co-reference relation with the cataphoric pronoun. (iii) Third, we examine if cataphoric pronoun dependencies generate the same kind of ERP components as forward pronoun dependencies. As discussed above, previous studies (e.g., Osterhout and Mobley, 1995; Van Berkum et al., 2007; Xu et al., 2013; a.o.) examined forward dependencies. However, no ERP study has examined cataphoric dependencies where the pronoun precedes the antecedent.

We aimed to search for the neuronal correlates of the ASM by means of a technique that has an excellent temporal resolution and where the effects of the active search can be examined by looking directly at brain behavior.

## Materials and Methods

### Materials

Thirty-six experimental items were constructed in Dutch. These 36 items were distributed across four lists in a Latin Square design, which implies that each participant saw nine trials per condition. We decided on the relatively small number of trials per condition for a number of reasons: (a) The GMME effect has been quite reliable in the behavioral literature. Thus, we expect the size effect of the gender mismatch to be robust; (b) we would like to avoid reading fatigue as well as participant developing different processing strategies derived from the high number of proper names included in the items. Note that previous studies, which investigated the processing of coreference involving repeated nouns with the ERP technique, used a higher number of trials per condition for their experiments (i.e., 40 trials per condition; see for example, Swaab et al., 2004; Ledoux et al., 2007). However, the research questions of these studies and our initial question do not overlap, since these studies were examining word repetition-priming effects and the impact this factor had on the modulation of the N400 ERP component, whereas our interest lays in the process of coreference itself. The vast majority of ERP experiments in the field present every participant with 20–40 items per condition, but this is because the ERP effects that the experimenters are after are often rather small. Likewise, the use of a large number of trials is often connected to the fact that usually some trials are discarded due to artifacts or to the type of ERP component that the researchers are after, which might be different in size (see for example, Luck, 2005; Kaan, 2007 for further discussion of this specific issue).

We followed closely the set-up of the English word-by-word self-paced reading experiment by Kazanina et al. (2007) while creating our ERP experiment, since we were interested in seeing the time-course of the GMME using ERPs. There are four experimental conditions, as shown in (7). First, No-Constraint conditions, which contain a possessive pronoun, in masculine (7a) or feminine form (7b) that matches or mismatches, respectively, in gender with the linearly first antecedent *Lodewijk* (masculine). Second, Principle C conditions, which contain a cataphoric nominative pronoun in masculine (7c) or feminine form (7d) that cannot co-refer with the referential expression *Lodewijk* in the embedded clause due to Principle C.

In all conditions, the test sentences always contain a licit antecedent for the pronoun. For example, in the No-Constraint mismatch condition in (7b) and in Principle C conditions in (7c) and (7d), the pronouns corefer with an antecedent that appears toward the end of each sentence [i.e., *Mirjam* in (7b) and (7d), and *Thomas* in (7c)]. Relevantly, even if pronouns could have co-reference with an antecedent outside of the sentence, the availability of an antecedent in the same sentence (i.e., *Mirjam/Thomas*) guarantees that the pronoun-antecedent relation is resolved within the sentence. Feminine and masculine pronouns and referential expressions were counter-balanced. Previous reading time studies found effects at positions immediately following the antecedent (see Yoshida et al., 2014). Based on this, we included proper names with a surname (such as *Lodewijk Boer*) in our data to ensure that there could be a region immediately following the proper name that was still connected to the antecedent position. However, considering the superior time accuracy of the ERP technique, our prediction was that the effect should be observable at the target position rather than at immediately following regions. Participants read 36 target stimuli such as those in (7; see Data Sheet in Supplementary Material for a whole list of stimuli) randomly interspersed with 35 unrelated fillers that were part of a different experiment that examined the processing of backward negative polarity item dependencies (Pablos et al., 2012).

(7) **a. No-Constraint/Match**
    **Zijn_**j**_** assistenten kwamen erachter dat **Lodewijk_**j**_ Boer** geen prijswinnaar *His assistants found out that Lodewijk_masc_ Boer no prizewinner* geselecteerd had, maar **Mirjam_**i**_** had geen interesse in de roddel.
    *selected had but Mirjam_fem_ had no interest in the gossip* ‘His assistants found out that Lodewijk Boer had not selected a prizewinner, but Mirjam had no interest in the gossip.’
    **(b) No-Constraint/Mismatch**
    **Haar_**i**_** assistenten kwamen erachter dat **Lodewijk_**j**_ Boer** geen prijswinnaar *Her assistants found out that Lodewijk_masc_ Boer no prizewinner* geselecteerd had, maar **Mirjam_**i**_** had geen interesse in de roddel. *selected had, but Mirjam_fem_ had no interest in the gossip.*
    ‘Her assistants found out that Lodewijk Boer had not selected a prizewinner, but Mirjam had no interest in the gossip.’
    **c. Principle C/Match**
    **Hij_**i**_** kwam erachter dat **Lodewijk_**j**_ Boer** geen prijswinnaar
    He found out that Lodewijk_masc_ Boer no prize winner
    geselecteerd had, maar **Thomas_**i**_** had geen interesse in de roddel.
    selected had, but Thomas_masc_ had no interest in the gossip.
    ‘He found out that Lodewijk Boer had not selected a prizewinner, but Thomas had no interest in the gossip.’
    **d. Principle C/Mismatch**
    **Zij_**i**_** kwam erachter dat **Lodewijk_**j**_ Boer** geen prijswinnaar
    She found out that Lodewijk_masc_ Boer no prize winner
    geselecteerd had, maar **Mirjam_**i**_** had geen interesse in de roddel.
    selected had, but Mirjam_fem_ had no interest in the gossip.
    ‘She found out that Lodewijk Boer had not selected a prizewinner, but Mirjam had no interest in the gossip.’

### Participants

Twenty-four students of Leiden University participated in this study, which was conducted at the EEG Laboratory in the Faculty of Social Sciences of Leiden University. They were all native speakers of Dutch. All participants had normal or corrected-to-normal vision, were right-handed, gave informed consent and were paid €12.50 for their participation, which lasted around 30 min, excluding set-up time. The experiment followed the Ethics Committee regulations of the Faculty of Social Sciences of Leiden University, which approved its implementation.

### Procedure

Participants were comfortably seated in a dimly lit testing room around 100 cm in front of a computer monitor. Sentences were presented one word at a time in black letters on a white screen using the presentation software E-prime (Psychology Software Tools Inc.). Each sentence was preceded by a fixation cross (“+”) which appeared at the center of the screen and remained there for 1000 ms. The fixation point was followed by a blank screen interval of 300 ms, and then the sentence was displayed word by word.

Each word appeared on the screen for 300 ms, followed by a fixation cross (“+”) at the center of the screen that remained visible for 300 ms. Participants were instructed to read the sentences carefully for comprehension. The last word of each sentence was marked with a period, and 1000 ms later a comprehension question appeared and prompted the participant to press a button to continue. Every experimental item was followed by a comprehension question. The comprehension questions targeted different positions of the sentence and some of them targeted the referential expressions *Lodewijk Boer* or *Thomas/Mirjam*. The comprehension questions were counter-balanced for yes and no answers and, for some items, they differed across conditions (see Data Sheet in Supplementary Material). Four counterbalanced lists derived from a Latin Square Design were used for the experiment. Before starting the experimental phase, eight warm-up practice trials were presented to the participants, which had no similarity to any of the targets or filler items in the experiment. Participants were able to ask clarification questions to the experimenter about the task at the practice time. The experimental session was broken up by two break periods, with a different number of items distributed across each block, with 35 and 36 sentences per block.

#### EEG Recording

The EEG signal was continuously acquired at a sampling frequency of 512 Hz using a BioSemi (Active Two) system from 32 Ag/AgC1 electrodes distributed in the scalp following the extended 10–20 convention (Fp1/2, FC5/, AF3/4, Fz, CP5/6, CP1/2, Cz, F7/8, F3/4, T7/8, C3/4, Pz, FC1/2, P3/4, O1/2, Oz, P7/8, PO3/4). EEG data was referenced on-line to two auxiliary electrodes: common mode sense (CMS) and driven right leg (DRL) and re-referenced off-line to the mean activity at the two mastoids. A high-pass filter with a cut-off frequency of 0.1 Hz was applied online to eliminate DC drifts. Vertical and horizontal eye movements were monitored with two electrodes at the infraorbital and supraorbital, and electrodes at the outer canthus of the right and left eyes. Electrode impedances were monitored during installation to ensure a low level of electronic noise.

#### EEG Analysis

For every subject, recorded EEG waveforms were post-processed before analysis to reduce noise and artifacts as much as possible. After applying a high-pass filter to remove slow drifts and DC offsets, ocular correction was performed using an implementation of the Gratton et al. (1983) algorithm. Other artifacts were removed both by visual inspection and by performing an automated detection based on gradient change rate. The process resulted in the rejection of 6% of the trials (51 out of 864) distributed among the experimental conditions as follows: (7a) 1%; (7b) 1%; (7c) 2%; (7d) 2%. To confirm that these small differences between conditions were not significant and did not introduce biases in the results, we ran a repeated measures mixed-logit analysis with Match (match/mismatch) and Constraint (No Constraint/Principle C) as independent variable and Subject as random factor. Both main effects and interactions were considered, and no significant difference in likelihood ratio between the fitted model and a null intercept only model was observed.

As a final step, a low-pass filter with a cut-off frequency of 30 Hz was applied to remove noise and non-neurological signals. After the data cleaning, a few electrodes identified as noisy or with intermittent connection were replaced by an interpolation based on neighboring channel responses.

Electroencephalography recordings were then segmented from 200 ms before to 800 ms after the onset of the significant region being analyzed (*Lodewijk*). A baseline correction was applied based on the average of the 200 ms prior to the stimulus onset.

Previous studies that have examined gender mismatches consistently reported a P600 component. In order to evaluate the presence of a P600 in our experimental data, the 500–700ms time window was tested by means of a 4-way repeated-measure ANOVA, considering four within-subject factors. Two to evaluate the signal scalp distribution: *Hemisphere* [Left *(Fp1, F3, F7, C3, P3, O1)* Central *(Fz, Cz, Pz)*, Right *(Fp2, F4, F8, C4, P4, O2)*], and *Position* [Frontal *(Fp1, Fp2, F3, F4, F7, F8)*, Medial *(C3, Cz, C4)*, and Parietal *(P3, Pz, P4, O1, O2)*]; and two to examine effects between conditions: *Constraint* (No Constraint/Principle C), and *Match* (Match/Mismatch). Mean voltage-amplitude was considered as the dependent variable in the analysis, and p-values where corrected for sphericity where required.

## Results

### Comprehension Questions

Average accuracy rates were high and no participants were rejected on the basis of accuracy (*M* = 84.59%, *SD* = 5.44%). The accuracy scores were similar across conditions (*M*_NoConstraintMatch_ = 81%, *M*_NoConstraintMismatch_ = 84%, *M*_PrincipleCMatch_ = 87%, *M*_PrincipleCMismatch_ = 86%). The difference in mean values was not significant as shown by a 2 × 2 repeated-measures ANOVA randomized by subjects with *Constraint* and *Match* as independent factors and Response Accuracy as dependent variable (*p* > 0.5 for all main effects and interactions).

### Event Related Potentials

**FIGURE 1:**
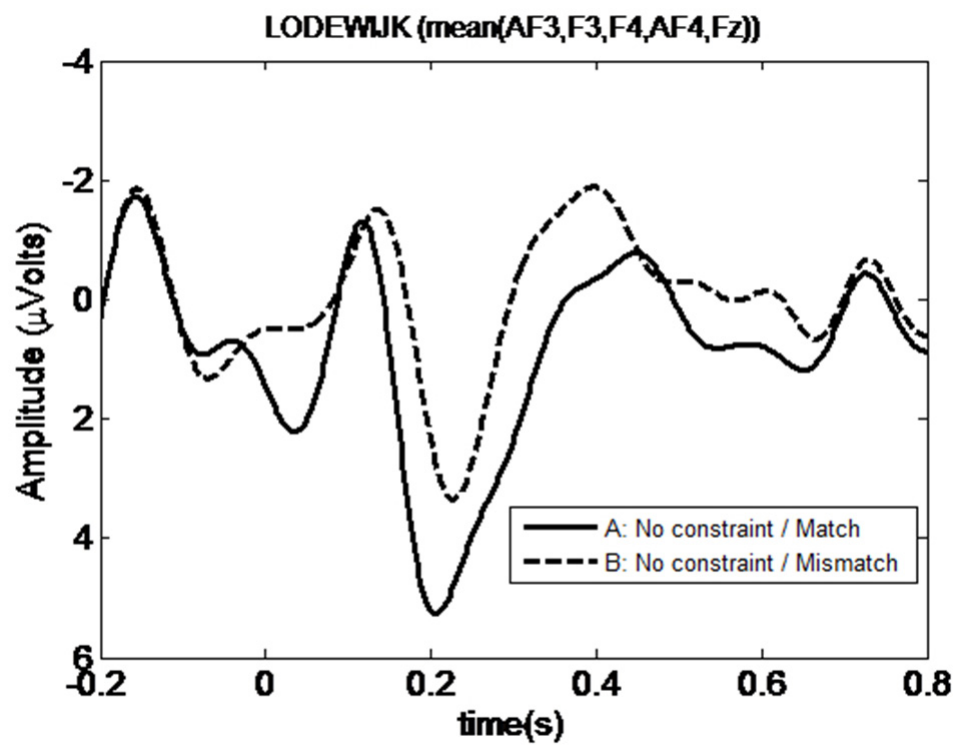
**Mean waveform at first potential antecedent *Lodewijk* position for *No-constraint Match* and *Mismatch* conditions**.

**FIGURE 2:**
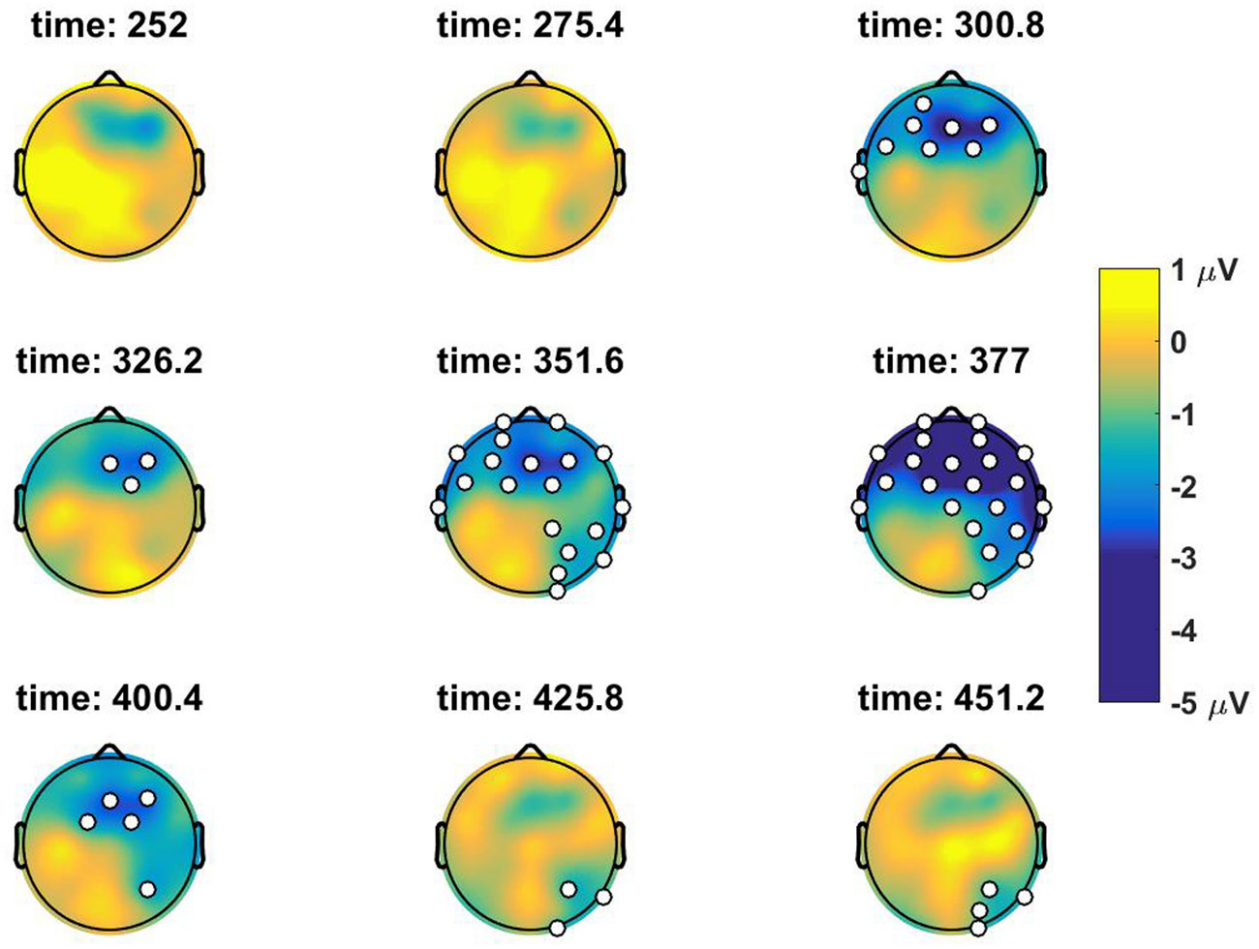
**Topographic scalp maps of the difference wave between the *No-Constraint Mismatch* and the *No-Constraint Match* condition at a series of discrete time positions.** The electrodes that were significantly different between the two conditions in the cluster mass univariate analysis (*p* < 0.07) are marked in white.

**FIGURE 3:**
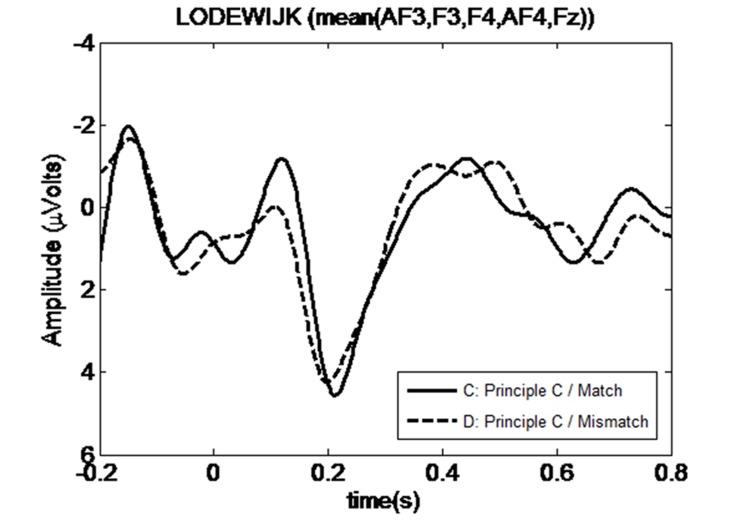
**Mean waveform at first potential antecedent *Lodewijk* position for *Principle C Match* and *Mismatch* conditions**.

**FIGURE 4:**
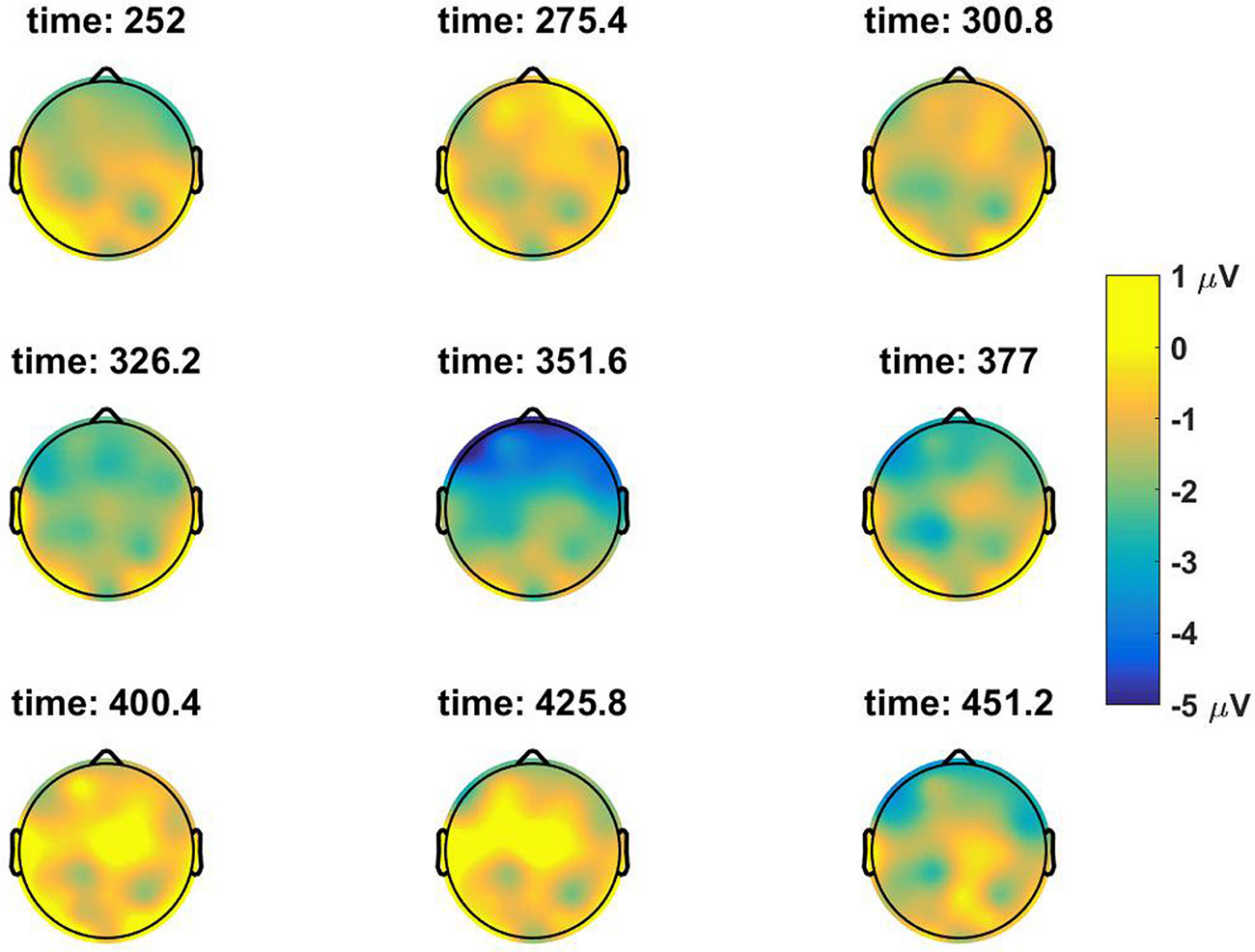
**Topographic scalp maps of the difference wave between the *Principle-C Mismatch* and the *Principle-C Match* condition at a series of discrete time positions**.

We investigated ERPs at the subject position of the embedded clause, *Lodewijk*, which is the first potential antecedent position in the sentence if there is no Principle C violation. Four-way ANOVA performed in the pre-selected P600 time window (500–700 ms) did not result in any significant main effect or interaction (*p* ≥ 0.1 in all cases), as shown in the right most column of **Table 1**. However, visual comparison of the grand average time traces in the anterior electrodes for the No-Constraint Mismatch condition (7b) versus No-Constraint Matched (7a) condition shows an apparent sustained negativity in the 200–600 ms region (**Figure 1**). The anterior topography of the negativity can be observed in **Figure 2**. No such negativity is observed for Principle C Match/Mismatch conditions (**Figures 3** and **4**). The asymmetry observed in the No-Constraint with respect to the Principle C conditions supports the expectation of the experimental manipulation, therefore, an exploratory analysis was performed to investigate the reliability and nature of this apparent difference.

**Table 1:** Multiple window ANOVA 4-way interaction results (*p*-values reported).

	Time window (ms)				P600 time window
	100–300	200–400	300–500	400–600	500–700 ms
Hemisphere × Position × Match × Constraint	0.085	0.037^∗^	0.036^∗^	0.069	0.101

*^∗^p < 0.05.*

An omnibus ANOVA performed in the complete 200–600 ms time window shows a significant 4-way interaction of *Constraint, Match, Hemisphere*, and *Position* [*F*(4,92) = 2.572; *p* = 0.043]. Follow-up simple interaction analysis for each level of the *Constraint* factor reveals no significant interaction or main effect in Principle C conditions, while a significant 3-way interaction between *Hemisphere × Match × Position* is present in No-Constraint [*F*(4,92) = 3.202, *p* = 0.016]. A further breakdown of this interaction for every level of the *Position* condition shows a significant effect of *Match* factor at the Anterior sites [*F*(1,23) = 4.82, *p* = 0.038], and no dependence on *Hemisphere*. The *No-Constraint Mismatch* condition (7b) waveform average amplitude is more negative than (7a) [*t*-test nearly significant difference *t′*(23) = 1.989, *p* = 0.057].

The same analysis was repeated using sliding 200 ms long windows to localize the effect with respect to the onset time of the stimuli. **Table 1** summarizes the omnibus ANOVAs and **Table 2** provides the follow up simple interaction evaluation for those regions with significant interaction in the omnibus ANOVA. (Only significant comparisons and effects are shown for readability. Values are corrected for sphericity where required – corrected *p*-values are reported).

**Table 2:** Simple interactions follow-up.

		Time window (ms)	
		200–400	300–500
Principle C	Match	0.428	0.813
	Hemisphere × Match	0.834	0.653
	Position × Match	0.120	0.127
	Match × Hemisphere × Position	0.096	0.288
No-Constraint	Match	0.085	0.185
	Hemisphere × Match	0.129	0.169
	Position × Match	0.071	0.072
	Match × Hemisphere × Position	0.013^∗^	0.018^∗^

*^∗^p < 0.05.*

Finally, **Table 3** shows a summary of the main effects and *post hoc* pairwise comparisons observed in the two time windows (200–400 ms, 300–500 ms) in the breakdown of the interactions observed in **Table 2**, which in all cases reflect a significant anterior negativity of the *Mismatch* condition for the *No-Constraint* case when compared with the *matched* counterpart.

**Table 3:** Simple comparisons “No Constraint” condition.

		Time window (ms)	
No constraint		200–400	300–500
Anterior	Match	0.011^∗^ *t* (23) = 2.52, *p* = 0.019	0.025^∗^ *t* (23) = 2.22, *p* = 0.036
	Hemisphere × Match	0.061	0.053
Central	Match	0.202	0.333
	Hemisphere × Match	0.409	0.219
Posterior	Match	0.498	0.643
	Hemisphere × Match	0.074	0.109

*^∗^p < 0.05.*

However, the results of the exploratory analysis above present the multiple comparison problem (MCP). To limit the Family Wise Error Rate (FWER) to a 5% level, the individual comparisons reported in **Table 1** should have a *p*-value lower than 0.05/4 = 0.0125. In addition, an individual 2 × 2 ANOVA – to verify the interaction of the *Constraint* and *Match* factors in the topographical regions of interest defined by the *Position* and *Hemisphere* factors considered in the above analysis – did not yield a significant interaction in neither of the time windows (*p* > 0.10). This result is very likely due to the low statistical power provided by the small number of electrodes in each region of interest, and the limited number of trials.

To address the problem of MCP and verify if the differences observed were reliable, the ERPs measured were analyzed with a repeated measures two-tailed cluster mass permutation test (Bullmore et al., 1999; Maris and Oostenveld, 2007) using the Matlab Mass Univariate ERP Toolbox (Groppe et al., 2011). This test provides a better spatial and temporal resolution and weak control of the FWER. We included all samples between 200 and 800 ms at all 32 electrodes. Electrodes within an approximate distance of 5.77 cm from each other were considered spatial neighbors for the cluster determination. Repeated measures *t*-tests were performed on the difference wave of the Match and Mismatch conditions for both *No-Constraint* and *Principle C* factor levels. *T*-test included the original data and 2500 random within-subjects permutations. With this technique, we tested separately the null hypothesis that the *Match* and *Mismatch* position do not differ in the No-Constraint and Principle C conditions. The maximum cluster-level mass procedure in the No-Constraint Match versus Mismatch comparison returned a cluster at the central-frontal electrodes extending temporally from 300 to ∼420 ms with an alpha level *p* = 0.07 (see **Figure 2**). In contrast, the procedure in the Principle C conditions did not reject the null hypothesis to any level of significance (*p* > 0.4).

In conclusion, results show significant differences to an alpha level of ∼0.07 between the *Match* and *Mismatch* conditions in the No-Constraint cases only, with anterior topographic distribution over a window around 300–420 ms. The observed difference is both in the direction expected based on the theoretical predictions, and with a coherent spatial and temporal localization. This reinforces that the effect is reliable even with the aforementioned reduced confidence level, compared to traditional 5% values. The presence of a positive result in an experiment with a relatively low power in terms of the number of trials observed per subject and condition (i.e., 9) suggests that the effect size is large and would be more prominent with an increased number of items [see Maxwell et al. (2008) for a discussion on sample size and statistical power].

## Discussion

### Active Search for Antecedents

We have shown that, in cases such as (7b) (No-Constraint Mismatch), where there is a gender mismatch between the pronoun and the first potential antecedent for this pronoun, an anterior negativity is generated at the potential antecedent position *Lodewijk*. This is not the case for (7a), where the potential antecedent matches in gender with the preceding pronoun. The anterior negativity could be interpreted as a result of the gender mismatch between a cataphoric pronoun and its antecedent, as well as the effect of failing to find an antecedent at the first potential position. However, for (7c) and (7d), where the cataphoric pronoun cannot corefer with the referential expression *Lodewijk* due to Principle C, no component is generated at the referential expression position. This confirms our predictions that (i) an active search for an antecedent is initiated as soon as a cataphoric pronoun is processed and that, (ii) although the ASM can be automatically initiated for every pronoun, which referential expression will be considered by the ASM is constrained by grammatical principles (in this case, Principle C). This result is in line with the behavioral results (e.g., Kazanina et al., 2007) that found a GMME at the potential antecedent.

### Forward vs. Backward Antecedent/Pronoun Dependencies and Prediction Failure

The differences observed in ERP components generated between our results in the case of cataphoric dependencies (anterior negativity) and the forward pronominal dependency studies (Osterhout and Mobley, 1995; Van Berkum et al., 2007; Xu et al., 2013; P600) raise questions on the nature of the effect observed.

In the current experiment, we focus on the relation between a *cataphoric* pronoun and its potential antecedent. In the case of forward antecedent-pronoun dependencies [as in (6)], there is no need to search for a pronoun after encountering the antecedent (e.g., *the aunt*) since this referential expression can be independently interpreted. In other words, we do not expect an active search for a pronoun in the case of forward dependencies. The P600 component in these cases, therefore, must correspond to a gender mismatch between the referential expression and the pronoun.

In backward, cataphoric pronoun-antecedent dependencies, on the other hand, the processes underlying the generation and interpretation of these dependencies are different since the interpretation of the pronoun needs to be resolved. It is therefore reasonable to hypothesize that the parser prefers to start a search as soon as a pronoun is encountered. The anterior negativity in our experiment could be interpreted as related to the searching process itself, namely, a failure of a prediction and not so much to the gender mismatch. The GMME provides the evidence that the antecedent search is active in the no-constraint cases, but it might not be the primary reason for the generation of the anterior negativity. Nevertheless, after having examined previous literature on gender mismatches, we might still wonder why no P600 as well is generated for the gender mismatch at *Lodewijk* in (7b) after encountering the feminine pronoun *haar*. We hypothesize that, in forward dependencies, the parser needs to retrieve the gender of the antecedent from memory and check for gender matching. The P600 could be a reflection of the gender mismatch alone. Conversely, in backward dependencies, the parser anticipates the appearance of an antecedent in the upcoming sentence as soon as it processes the pronoun. Thus, when the parser encounters the first potential antecedent position, it expects to find a matched antecedent. When it fails, there is a negativity generated instead of a P600 because the failure of finding a matching antecedent prevails over the GMME. With this claim we do not intend to imply that the gender mismatch does not occur at all or that it does not precede the expectation failure (since the failure of the prediction cannot occur before the mismatch is detected) rather that the failure of finding a matching antecedent veils the presence of a P600.

In the second experiment in Osterhout and Mobley (1995), a negativity (at anterior and temporal sites in the left-hemisphere between 300 and 500 ms) is found for a dependency where a specific verb form that agrees with the subject is predicted and fails. In our experiment, a negativity is found for a dependency where an antecedent for the cataphoric pronoun is predicted and this prediction fails because of a gender mismatch. These two types of dependencies are different in nature (one involves subject-verb agreement and the other a pronoun-antecedent coreferential relation), but the mechanism of prediction failure seems to be the same in that there is a negative component generated in both cases. Despite of the fact that the negativities in these two studies are different in distribution, we suggest that they are connected to the same basic process, and that they reflect the failure of a previously established expectation. However, we have to consider that the presence of a negativity in agreement violations is currently under debate since not all the studies observed it (see Nevins et al., 2007; Mancini et al., 2011; Molinaro et al., 2011; a.o.).

### Potential Task and Stimuli Presentation Effects

One of the potential sources for the lack of P600 for the gender mismatch in our study might connect to issues that previous studies have discussed (Bornkessel-Schlesewsky et al., 2011; Molinaro et al., 2011; Sassenhagen et al., 2014), such as the influence of task and the modality of stimulus presentation. The current study used word-by-word visual presentation of the sentences in which subjects had to read the sentence and answer a Yes/No comprehension question afterward. Studies that have shown P600 effects for gender mismatches in forward antecedent/pronoun dependencies (Osterhout and Mobley, 1995; Van Berkum et al., 2007; Xu et al., 2013) have all used visual presentation, so the mode of presentation does not seem to have an impact in the results. Differences between our study and previous studies rest in the task that participants were required to complete. Van Berkum et al. (2007) do not require any task from participants besides reading the sentences, whereas Osterhout and Mobley (1995) and Xu et al. (2013) ask their participants to conduct an acceptability judgment after reading each sentence. Sassenhagen et al. (2014) discuss the idea that the generation of a P600 can be task-dependent and that consciously detected violations might differ with respect to non-consciously detected violations in that the detected or attentive violations elicit both an early negative component and a P600, whereas the non-detected ones do not necessarily elicit a P600 (Hasting and Kotz, 2008; Batterink and Neville, 2013). Results from our experiment seem to align with this idea since we only get an early negativity and the study does not implement a task that highlights the mismatch.

### Temporal Characteristics and Scalp Distribution of Negativities in Previous ERP Studies

Previous studies that have elicited negativities have looked at agreement mismatches with personal pronouns and subject-verb agreement failures (Osterhout and Mobley, 1995), at noun phrases that ambiguously referred to two equally suitable referents (Van Berkum et al., 2003, 2007), at incorrect cases of noun ellipsis (Martin et al., 2012), at pronoun and verb-agreement violations (Coulson et al., 1998), at verb subcategorization violations (Rösler et al., 1993), at phrase structure violations (Neville et al., 1991; Osterhout and Holcomb, 1992) and at conditions of increased memory load (Kluender and Kutas, 1993; King and Kutas, 1995; Friederici et al., 1996; Müller et al., 1997; Münte et al., 1998; Fiebach et al., 2001).

All the negativities found in these studies reflect syntactic processes and in many cases they represent a response to syntactic violations. However, they do not always have the exact same scalp distribution or topography as the negativity in our study. Osterhout and Mobley (1995) tested agreement mismatches involving personal pronouns in forward dependencies in their first experiment (discussed under the section on ERP Studies on Gender Agreement/Mismatch in the introduction) and found that a small sample of participants (*N* = 4) who judged the sentence as grammatical (and thus considered that there was an antecedent outside the clause for the pronoun) showed a sustained negativity in frontal electrodes in the 500–800 ms. The referentially induced frontal negativity (Nref) elicited by Van Berkum et al. (2003, 2007) was a widely distributed and frontally sustained negativity, emerging at about 300–400 ms after their acoustic onset, whereas Martin et al. (2012)’s negativity had a broad central distribution and emerged between 400 and 1000 ms after word onset. In Coulson et al. (1998), the negativity elicited by ungrammatical pronouns was largest at left anterior sites while that elicited by ungrammatical verbs was centro-parietal and slightly larger over the right hemispheres. This effect was largest between 300 and 500 ms after stimulus onset. ERPs for syntactic violations in Rösler et al. (1993) were negative between 400 and 700 ms after target onset and were more pronounced at anterior sites and over the left hemisphere. In Neville et al. (1991), the phrase structure violations generated a negative response between 300 and 500 ms over temporal and parietal regions of the left hemisphere while in Osterhout and Holcomb, 1992, the negativity occurred between 300 and 500 ms post stimulus at left hemisphere anterior sites.

If we look at the studies with increased memory load, the sustained negativity in Fiebach et al. (2001) started at about 400 ms after the onset of the first prepositional phrase and was maximal at left-anterior electrode positions. Friederici et al. (1996) found a left anterior negativity for the syntactic-category violation condition in auditory and visual tasks in the time windows between 400 and 600 ms (for auditory) and 350 and 500 ms (for visual) after word onset. The ERPs to the verbs in Object relative clause sentences (i.e., *The reporter who the senator harshly attacked admitted the error*) in King and Kutas (1995) showed more prolonged negativity over left anterior regions of the scalp than those in Subject relative clause sentences (i.e., *The reporter who harshly attacked the senator admitted the error*), and in Kluender and Kutas (1993), a difference was seen in the ERP between 300 and 500 ms. post stimulus when wh-questions were compared to yes/no questions at a position early in the matrix clause. Finally, in Müller et al. (1997), there was a large fronto-central negativity beginning at the gap in the Object relative clause sentences and a left frontal negativity in Münte et al. (1998).

### Referential Dependencies that Generated Negativities in Previous ERP Studies

Among the ERP studies that have generated negativities, Martin et al. (2012) report a centrally distributed negativity at a position that renders a gender-mismatch effect [i.e., the determiner *otro* ‘another (MASC)’], which mismatches in gender with the antecedent *camiseta* [‘t-shirt (FEM)’] in cases of noun ellipsis in coordinated sentences. In their study, the gender mismatch results in an ungrammatical sentence (its interpretation cannot be recovered, unlike in (7b) in our study where a second potential antecedent *Mirjam* can be used to resolve the interpretation of the pronoun *haar*) and the position in which the mismatch is detected is a determiner that allows nominal ellipsis within the second coordinated sentence. Both Martin et al. (2012) and our study examine the resolution of dependencies where a referential entity and an antecedent are involved and both concern gender mismatches. However, similar to the first experiment on the study in Osterhout and Mobley (1995) on forward pronominal dependencies, in Martin et al.’s (2012) study, the interpretation of a determiner that allows nominal ellipsis and whose antecedent sits in the previous coordinated clause might involve a completely different process from the process required in the dependencies examined within the current study, since the antecedent does not necessarily start a search for the determiner in the second conjunct.

A sustained negativity (largest at anterior sites) has additionally been found in cases of referential ambiguity under the name of referentially induced frontal negativity (Nref; Van Berkum et al., 2003, 2007), where participants had to choose among a set of equally plausible referents for a specific noun phrase. The fact that Van Berkum et al. (2003, 2007) and our study both cover the processing of dependencies that involve referential expressions, might have contributed to the overlapping characteristics of the ERP components that were found.

In short, we have argued that the anterior negativity in this study can be connected to negativities found in previous studies in that it involves (1) a gender mismatch; (2) a dependency that contains referential expressions in which coreference needs to be established, and (3) a dependency in which an expectation of the parser fails. Thus, even if the studies discussed thus far have looked at different phenomena, it seems that there are some common processes underlying all these negativities, such as building a referential dependency on-line and predicting a specific upcoming element in the sentence.

## Conclusion

In our ERP study on the processing of cataphoric pronoun dependencies in Dutch, we replicated earlier behavioral findings (Sturt, 2003; Van Gompel and Liversedge, 2003; Kazanina et al., 2007; Yoshida et al., 2014) supporting that the parser actively looks for an antecedent for a cataphoric pronoun in the upcoming sentence (even when this pronoun could have coreference with an antecedent outside of the sentence), but restricts its choice to grammatically licit positions. This is evidenced by the fact that no ERP effect is elicited at the potentially mismatched referential expression in the conditions where Principle C of the Binding Theory bars coreference. The overall results show that the GMME connected to longer reading times in previous behavioral experiments is reflected in the current ERP study as an anterior negativity elicited at the potential antecedent in cataphoric dependencies. We postulate that this anterior negativity reflects the prediction failure for an appropriate antecedent after encountering a sentence initial pronoun.

## Conflict of Interest Statement

The authors declare that the research was conducted in the absence of any commercial or financial relationships that could be construed as a potential conflict of interest.
