# Supplementary material for: Active search for antecedents in cataphoric pronoun resolution
Source: Front Psychol. 2015 Oct 30;6:1638. doi: 10.3389/fpsyg.2015.01638 (PMC4627476; doi:10.3389/fpsyg.2015.01638)
Supplement: Supplementary file 1 [file Data_Sheet_1.PDF]

Experimental Items

Pablos L. , Doetjes J., Ruijgrok B. , Cheng L.
Active Search for Antecedents in Cataphoric Pronoun Resolution

a. No Constraint / Match
b. No Constaint / Mismatch
c. Principle C / Match
d. Principle C / Mismatch

1 a
Zijn assistenten kwamen erachter dat Lodewijk Boer geen prijswinnaar geselecteerd had, maar Mirjam had geen interesse in roddels.

? Is het waar dat er geen prijswinnaar geselecteerd was? Y

1 b
Haar assistenten kwamen erachter dat Lodewijk Boer geen prijswinnaar geselecteerd had, maar Mirjam had geen interesse in roddels.

? Is het waar dat er geen prijswinnaar geselecteerd was? Y

1 c
Hij kwam erachter dat Lodewijk Boer geen prijswinnaar geselecteerd had, maar Thomas had geen interesse in roddels.

? Is het waar dat er geen prijswinnaar geselecteerd was? Y

1 d
Zij kwam erachter dat Lodewijk Boer geen prijswinnaar geselecteerd had, maar Thomas had geen interesse in roddels.

? Is het waar dat er geen prijswinnaar geselecteerd was? Y

2 a
Zijn managers maakten bekend dat Maarten Bakker een script geselecteerd had, maar Annie leek hier niet in geïnteresseerd.

? Werd geheim gehouden dat Maarten Bakker een script geselecteerd had? N

2 b
Haar managers maakten bekend dat Maarten Bakker een script geselecteerd had, maar Annie leek hier niet in geïnteresseerd.

? Werd geheim gehouden dat Maarten Bakker een script geselecteerd had? N

2 c
Hij maakte bekend dat Maarten Bakker een script geselecteerd had, maar Eddie leek hier niet in geïnteresseerd.

? Werd geheim gehouden dat Maarten Bakker een script geselecteerd had? N

2 d
Zij maakte bekend dat Maarten Bakker een script geselecteerd had, maar Annie leek hier niet in geïnteresseerd.

? Werd geheim gehouden dat Maarten Bakker een script geselecteerd had? N

3 a
Zijn secretaresses maakten duidelijk dat Adriaan Kist geen datum geprikt had, maar Jenny verwachtte gauw uitsluitsel te krijgen.

? Maakten de secretaresses duidelijk dat er geen datum peprikt was? Y

3 b
Haar secretaresses maakten duidelijk dat Adriaan Kist geen datum geprikt had, maar Jenny verwachtte gauw uitsluitsel te krijgen.

Q Maakten de secretaresses duidelijk dat er geen datum peprikt was? Y

3 c
Hij maakte duidelijk dat Adriaan Kist geen datum geprikt had, maar Boris verwachtte verwacht-te gauw uitsluitsel te krijgen.

? Werd duidelijk gemaakt dat Adriaan Kist geen datum geprikt had? Y

3 d
Zij maakte duidelijk dat Adriaan Kist geen datum geprikt had, maar Jenny verwachtte gauw uit-sluitsel te krijgen.

Q Werd duidelijk gemaakt dat Adriaan Kist geen datum geprikt had? Y

4 a
Zijn zaakwaarnemers waren zeker dat Joachim Bakker de politie geïnformeerd had, maar Alida wist verder geen smeuïge details.

? Informeerde Joachim de brandweer? N

4 b
Haar zaakwaarnemers waren zeker dat Joachim Bakker de politie geïnformeerd had, maar Ali-da wist verder geen smeuïge details.

? Informeerde Joachim de brandweer? N

4 c
Hij was zeker dat Joachim Bakker de politie geïnformeerd had, maar Boris wist verder geen smeuïge details.

? Informeerde Joachim de brandweer? N

4 d
Zij was zeker dat Joachim Bakker de politie geïnformeerd had, maar Alida wist verder geen smeuïge details.

? Informeerde Joachim de brandweer? N

5 a
Haar collega's lieten weten dat Elsemiek Wieringa van fraude beschuldigd was, maar Kevin weigerde de bewering te geloven.

? Geloofde Kevin de bewering over Elsemieke? N

5 b
Zijn collega's lieten weten dat Elsemiek Wieringa van fraude beschuldigd was, maar Kevin weigerde de bewering te geloven.

? Geloofde Kevin de bewering over Elsemieke? N

5 c
Zij liet weten dat Elsemiek Wieringa van fraude beschuldigd was, maar Karin weigerde de bewering te geloven.

? Geloofde Karin de bewering over Elsemieke? N

5 d
Hij liet weten dat Elsemiek Wieringa van fraude beschuldigd was, maar Kevin weigerde de bewering te geloven.

? Geloofde Kevin de bewering over Elsemieke? N

6 a
Haar secretaresses merkten op dat Annabel Feijts een fout gemaakt had, dus Mark nam direct de nodige maatregelen.

? Werden er maatregelen genomen door Mark? Y

6 b
Zijn secretaresses merkten op dat Annabel Feijts een fout gemaakt had, dus Mark nam direct de nodige maatregelen.

? Werden er maatregelen genomen door Mark? Y

6 c
Zij merkte op dat Annabel Feijts een fout gemaakt had, dus Marie nam direct de nodige maatregelen.

? Werden er maatregelen genomen door Marie? Y

6 d
Hij merkte op dat Annabel Feijts een fout gemaakt had, dus Mark nam direct de nodige maatregelen.

? Werden er maatregelen genomen door Mark? Y

7 a
Haar vrienden zagen in dat Marianne Raaphorst huidige politieke ontwikkelingen bekritiseerde, maar Bart zag er geen logica in.

? Werd Marianne's kritiek op huidige politieke ontwikkelingen opgemerkt? Y

7 b
Zijn vrienden zagen in dat Marianne Raaphorst huidige politieke ontwikkelingen bekritiseerde, maar Bart zag er geen logica in.

? Werd Marianne's kritiek op huidige politieke ontwikkelingen opgemerkt? Y

7 c
Zij zag in dat Marianne Raaphorst huidige politieke ontwikkelingen bekritiseerde, maar Bea zag er geen logica in.

? Werd Marianne's kritiek op huidige politieke ontwikkelingen opgemerkt? Y

7 d
Hij zag in dat Marianne Raaphorst huidige politieke ontwikkelingen bekritiseerde, maar Bart zag er geen logica in.

? Werd Marianne's kritiek op huidige politieke ontwikkelingen opgemerkt? Y

8 a
Haar bewonderaars maakten duidelijk dat Caroline Huibregts stevig bekritiseerd geweest was, maar Floris was daar stiekempjes blij over.

? Was Floris bedroefd over het feit dat het TV-bedrijf werd bekritiseerd? N

8 b
Zijn bewonderaars maakten duidelijk dat Caroline Huibregts stevig bekritiseerd geweest was, maar Floris was daar stiekempjes blij over.

? Was Floris bedroefd over het feit dat het TV-bedrijf werd bekritiseerd? N

8 c
Zij maakte duidelijk dat Caroline Huibregts stevig bekritiseerd geweest was, maar Maria was daar stiekempjes blij over.
? Was Maria bedroefd over het feit dat het TV-bedrijf werd bekritiseerd? N

8 d
Hij maakte duidelijk dat Caroline Huibregts stevig bekritiseerd geweest was, maar Floris was daar stiekempjes blij over.

? Was Floris bedroefd over het feit dat het TV-bedrijf werd bekritiseerd? N

9 a

Zijn kantoorgenoten waren verbaasd dat Sjors Koenen plannen in ontwikkeling had, maar Marianne had er grote verwachtingen van.

? Ondernam men stappen tegen de plannen van Sjors Koenen ? N

9 b
Haar kantoorgenoten waren verbaasd dat Sjors Koenen plannen in ontwikkeling had, maar Marianne had er grote verwachtingen van.

? Ondernam men stappen tegen de plannen van Sjors Koenen ? N

9 c
Hij was verbaasd dat Sjors Koenen plannen in ontwikkeling had, maar Frederik had er grote verwachtingen van.

? Ondernam men stappen tegen de plannen van Sjors Koenen ? N

9 d
Zij was verbaasd dat Sjors Koenen plannen in ontwikkeling had, maar Marianna had er grote verwachtingen van.

? Ondernam men stappen tegen de plannen van Sjors Koenen ? N

10 a
Haar vrienden waren zeker dat Nicole Gruyter gedwongen werd te verhuizen, maar Andre besloot dat niet te melden.

? Zou Nicole binnenkort op een lange vakantie? N

10 b
Zijn vrienden waren zeker dat Nicole Gruyter gedwongen werd te verhuizen, maar Andre besloot dat niet te melden.

? Zou Nicole binnenkort op een lange vakantie? N

10 c
Zij was zeker dat Nicole Gruyter gedwongen werd te verhuizen, maar Sylvia besloot dat niet te melden.

? Zou Nicole binnenkort op een lange vakantie? N

10 d
Hij was zeker dat Nicole Gruyter gedwongen werd te verhuizen, maar Andre besloot dat niet te melden.

? Zou Nicole binnenkort op een lange vakantie? N

11 a
Zijn jaargenoten waren verbaasd dat Antonie Verschuuren een faillissement moest aanvragen, maar Sanne pretendeerde dit al te weten.

? Moest Antoine een faillissement aanvragen? Y

11 b
Haar jaargenoten waren verbaasd dat Antonie Verschuuren een faillissement moest aanvragen, maar Sanne pretendeerde dit al te weten.

? Moest Antoine een faillissement aanvragen? Y

11 c
Hij was verbaasd dat Antonie Verschuuren een faillissement moest aanvragen, maar Jannes pretendeerde dit al te weten.

? Moest Antoine een faillissement aanvragen? Y

11 d
Zij was verbaasd dat Antonie Verschuuren een faillissement moest aanvragen, maar Sanne pretendeerde dit al te weten.

? Moest Antoine een faillissement aanvragen? Y

12 a
Haar managers waren verbaasd dat Charlot Meijer met overheidsgeld gesponsord werd, maar Arthur dacht dat het niet uitmaakte.

? Was er verbazing over de bron van de sponsoring van het programma? Y

12 b
Zijn managers waren verbaasd dat Charlot Meijer met overheidsgeld gesponsord werd, maar Arthur dacht dat het niet uitmaakte.

? Was er verbazing over de bron van de sponsoring van het programma? Y

12 c
Zij was verbaasd dat Charlot Meijer met overheidsgeld gesponsord werd, maar Arthur dacht dat het niet uitmaakte.

? Was er verbazing over de bron van de sponsoring van het programma? Y

12 d
Hij was verbaasd dat Charlot Meijer met overheidsgeld gesponsord werd, maar Arthur dacht dat het niet uitmaakte.

? Was er verbazing over de bron van de sponsoring van het programma? Y

13 a
Zijn supporters hielden geheim dat Patrick Montagne altijd controversieel geweest was, maar Theresa moest het in ogenschouw nemen.

? Werd informatie over de controversiele statuur van Patrick achter gehouden? Y

13 b

Haar supporters hielden het geheim dat Patrick Montagne altijd controversieel geweest was, maar Theresa moest het in ogenschouw nemen.

? Werd informatie over de controversiele statuur van Patrick achter gehouden? Y

13 c
Hij hield het geheim dat Patrick Montagne altijd controversieel geweest was, maar Douwe moest het in ogenschouw nemen.

? Werd informatie over de controversiele statuur van Patrick achter gehouden? Y

13 d
Zij hield het geheim dat Patrick Montagne altijd controversieel geweest was, maar Theresa moest het in aanmerking nemen.

? Werd informatie over de controversiele statuur van Patrick achter gehouden? Y

14 a
Haar teamgenoten kondigden aan dat Suzanne Jansen zeer hoog gewaardeerd werd, maar Edward meldde niet de exacte waardering.

? Melde Edward de exacte waardering? N

14 b
Zijn teamgenoten kondigden aan dat Suzanne Jansen zeer hoog gewaardeerd werd, maar Edward meldde niet de exacte waardering.

? Melde Edward de exacte waardering? N

14 c
Zij kondigde aan dat Suzanne Jansen zeer hoog gewaardeerd werd, maar Monika meldde niet de exacte waardering.

? Melde Monika de exacte waardering? N

14 d
Hij kondigde aan dat Suzanne Jansen zeer hoog gewaardeerd werd, maar Edward melde niet de exacte waardering.

? Melde Edward de exacte waardering? N

15 a
Zijn buren waren onzeker dat Richard Valk aan de wet gehoorzaamde, maar Nienke stuurde toch de formulieren in.

? Was er onzekerheid over gehoorzaamheid aan de wet? Y

15 b
Haar buren waren onzeker dat Richard Valk aan de wet gehoorzaamde, maar Nienke stuurde toch de belastingformulieren in.

? Was er onzekerheid over gehoorzaamheid aan de wet? Y

15 c
Hij was onzeker dat Richard Valk aan de wet gehoorzaamde, maar Michael stuurde toch de belastingformulieren in.

? Was er onzekerheid over gehoorzaamheid aan de wet? Y

15 d
Zij was onzeker dat Richard Valk aan de wet gehoorzaamde, maar Nienke stuurde toch de belastingformulieren in.

? Was er onzekerheid over gehoorzaamheid aan de wet? Y

16 a
Haar sponsors wisten niet dat Karin Menken telefonisch te bereiken was, maar zou het niet opnieuw proberen.

? Zou Philip het opnieuw proberen? N

16 b
Zijn sponsors wisten niet dat Karin Menken telefonisch te bereiken was, maar Philip zou het niet opnieuw proberen.

? Zou Philip het opnieuw proberen? N

16 c
Zij wist niet dat Karin Menken telefonisch te bereiken was, maar Mirjam zou het niet opnieuw proberen.

? Zou Philip het opnieuw proberen? N

16 d
? Was Karen Manfred happy about the museum's art survey? N

Hij wist niet dat Karin Menken telefonisch te bereiken was, maar Philip zou het niet opnieuw proberen.

? Zou Philip het opnieuw proberen? N

17 a
Zijn collega's hielden stil dat Steven Monks misschien ontslagen zou worden, maar Laura vertelde wel het overige nieuws.

? Was het de familie die Steven's werksituatie stil hield? N

17 b
Haar collega's hielden stil dat Steven Monks misschien ontslagen zou worden, maar Laura vertelde wel het overige nieuws.

? Was het de familie die Steven's werksituatie stil hield? N

17 c
Hij hield stil dat Steven Monks misschien ontslagen zou worden, maar David vertelde wel het overige nieuws.

? Was het de familie die Steven’s werksituatie stil hield? N

17 d
Zij hield stil dat Steven Monks misschien ontslagen zou worden, maar Laura vertelde wel het overige nieuws.

? Was het de familie die Steven’s werksituatie stil hield? N

18 a
Zijn vrienden wisten niet dat William Slagwijn al lang geëmigreerd was, hoewel Sofie dit al een tijdje vermoedde.

? Was William Slagwijn geëmigreerd? Y

18 b
Haar vrienden wisten niet dat William Slagwijn al lang geëmigreerd was, hoewel Sofie dit al een tijdje vermoedde.

? Was William Slagwijn geëmigreerd? Y

18 c
Hij wist niet dat William Slagwijn al lang geëmigreerd was, hoewel Tom dit al een tijdje vermoedde.

? Was William Slagwijn geëmigreerd? Y

18 d
Zij wist niet dat William Slagwijn al lang geëmigreerd was, hoewel Sofie dit al een tijdje ver-moedde.

? Was William Slagwijn geëmigreerd? Y

19 a
Haar bondgenoten voerden aan dat Marjolein Davids de lokale verkiezingen boycotte, maar Erik zag af van aanvullend commentaar.

? Geloofde Erik dat Marjolein de lokale verkiezingen boycotte? N
? Leverde Erik verder commentaar? N
19 b
Zijn bondgenoten voerden aan dat Marjolein Davids de lokale verkiezingen boycotte, maar Erik zag af van aanvullend commentaar.

? Leverde Erik verder commentaar? N

19 c

Zij voerde aan dat Marjolein Davids de lokale verkiezingen boycotte, maar Jet zag zag af van aanvullend commentaar.

? Leverde Jet verder commentaar? N

19 d
Hij voerde aan dat Marjolein Davids de lokale verkiezingen boycotte, maar Erik zag af van aanvullend commentaar.

? Leverde Erik verder commentaar? N

20 a
Zijn investeerders waren zeker dat Rogier Putter door details misleid was, dus Kaat twijfelde of onderzoek nodig was.

? Waren de investeerders zeker dat Rogier Putter misleid was? Y

20 b
Haar investeerders waren zeker dat Rogier Putter door details misleid was, dus Kaat twijfelde of onderzoek nodig was.

? Waren de investeerders zeker dat Rogier Putter misleid was? Y

20 c
Hij was zeker dat Rogier Putter door details misleid was, dus Mark twijfelde of onderzoek nodig was.

? Werd het zeker geacht dat Rogier Putter misleid was? Y

20 d
Zij was zeker dat Rogier Putter door details misleid was, dus Kaat twijfelde twijfelde of onderzoek nodig was.

? Werd het zeker geacht zeker dat Rogier Putter misleid was? Y

21 a
Haar trainers kondigden aan dat Christine Luyt publiekelijk berispt zou worden, maar Bruno dacht dat onderzoek nodig was.

? Werd aangekondigd dat Cristine Luyt publiekelijk berispt zou worden? Y

21 b
Zijn trainers kondigden aan dat Christine Luyt publiekelijk berispt zou worden, maar Bruno dacht dat onderzoek nodig was.

? Werd aangekondigd dat Cristine Luyt publiekelijk berispt zou worden? Y

21 c
Zij kondigde aan dat Christine Luyt publiekelijk berispt zou worden, maar Maartje dacht dat onderzoek nodig was.

? Werd aangekondigd dat Cristine Luyt publiekelijk berispt zou worden? Y

21 d
Hij kondigde aan dat Christine Luyt publiekelijk berispt zou worden, maar Bruno dacht dat onderzoek nodig was.

? Werd aangekondigd dat Cristine Luyt publiekelijk berispt zou worden? Y

22 a
Haar managers vertelden niet dat Bettine Hoeks het stuk zou plaatsen, dus Jasper moest zorgvuldig te werk gaan.

? Moest Bettine Hoeks een urgente zaak oplossen? N
Must.pst Bettine Hoeks an urgent case solve (no, she should publish an article)

22 b
Zijn managers vertelden niet dat Bettine Hoeks het stuk zou plaatsen, dus Jasper moest zorgvuldig te werk gaan.

? Moest Bettine Hoeks een urgente zaak oplossen? N

22 c
Zij vertelde niet dat Bettine Hoeks het stuk zou plaatsen, dus Josien moest zorgvuldig te werk gaan.

? Moest Bettine Hoeks een urgente zaak oplossen? N

22 d
Hij vertelde niet dat Bettine Hoeks het stuk zou plaatsen, dus Jasper moest zorgvuldig te werk gaan.

? Moest Bettine Hoeks een urgente zaak oplossen? N

23 a
Haar ouders maakten duidelijk dat Margreet Ensink een rechtszaak zou aanspannen, maar Bert noemde niet een exacte datum.

? Zou Margreet een rechtszaak aanspannen? Y

23 b
Zijn ouders maakten duidelijk dat Margreet Ensink een rechtszaak zou aanspannen, maar Bert noemde niet een exacte datum.

? Zou Margreet een rechtszaak aanspannen? Y

23 c
Zij maakte duidelijk dat Margreet Ensink een rechtszaak zou aanspannen, maar Sjaan noemde niet een exacte datum.

? Zou Margreet een rechtszaak aanspannen? Y

23 d
Hij maakte duidelijk dat Margreet Ensink een rechtszaak zou aanspannen, maar Bert noemde niet een exacte datum.

? Zou Margreet een rechtszaak aanspannen? Y

24 a
Zijn accountants wisten zeker dat Antonie Smit een grote kaakoperatie onderging, maar Lies hield deze vervelende omstandigheid geheim.

? Zouden de accountants een kaakoperatie ondergaan? N

24 b
Haar accountants wisten zeker dat Antonie Smit een grote kaakoperatie onderging, maar Lies hield deze vervelende omstandigheid geheim.

? Zouden de accountants een kaakoperatie ondergaan? N

24 c
Hij wist zeker dat Antonie Smit een grote kaakoperatie onderging, maar Tim hield deze vervelende omstandigheid geheim.

? Zouden Antoine Smit een beenoperatie ondergaan? N

24 d
Zij wist zeker dat Antonie Smit een grote kaakoperatie onderging, maar Lies hield deze vervelende omstandigheid geheim.

? Zouden Zouden Antoine Smit een beenoperatie ondergaan? N

25 a
Zijn woordvoerders lieten weten dat Sebatiaan Bannink een stap terug deed, maar Jet wist niet het fijne ervan.

? Ging Sebastiaan Bannink extra werk doen? N

25 b
Haar woordvoerders lieten weten dat Sebatiaan Bannink een stap terug deed, maar Jet wist niet het fijne ervan.

? Ging Sebastiaan Bannink extra werk doen? N

25 c
Hij liet weten dat Sebatiaan Bannink een stap terug deed, maar Dirk wist niet het fijne ervan.

? Ging Sebastiaan Bannink extra werk doen? N

25 d
Zij liet weten dat Sebatiaan Bannink een stap terug deed, maar Jet wist niet het fijne ervan.

? Ging Sebastiaan Bannink extra werk doen? N

26 a
Zijn advocaten waren zeker dat Benjamin Soeter zwaar bestraft zou worden, maar Marie wist niet precies hoe zwaar.

? Zou Benjamin Soeter worden bestraft? Y

26 b
Haar advocaten waren zeker dat Benjamin Soeter zwaar bestraft zou worden, maar Marie wist niet hoe hoog de straf zou zijn.

? Zou Benjamin Soeter worden bestraft? Y

26 c
Hij was zeker dat Benjamin Soeter zwaar bestraft zou worden, maar Sjaak wist niet hoe hoog de straf zou zijn.

? Zou Benjamin Soeter worden bestraft? Y

26 d
Zij was zeker dat Benjamin Soeter zwaar bestraft zou worden, maar Marie wist niet hoe hoog de straf zou zijn.

? Zou Benjamin Soeter worden bestraft? Y

27 a
Haar ondergeschikten maakten bekend dat Marianne Welten frauduleuze praktijken ontdekt had, maar Sven betwijfelde of het waar was.

? Maakten men bekend dat Josine Welten financiele problemen had? N

27 b
Zijn ondergeschikten maakten bekend dat Marianne Welten frauduleuze praktijken ontdekt had, maar Sven betwijfelde of het waar was.

? Maakten men bekend dat Josine Welten financiele problemen had? N

27 c
Zij maakte bekend dat Marianne Welten frauduleuze praktijken ontdekt had, maar Sarah betwijfelde of het waar was.

? Maakte men bekend dat Josine Welten financiele problemen had? N

27 d
Hij maakte bekend dat Marianne Welten frauduleuze praktijken ontdekt had, maar Sven betwijfelde of het waar was.

? Maakte men bekend dat Josine Welten financiele problemen had? N

28 a

Zijn studenten kwamen erachter dat Joost Hagestein de bijeenkomst voorbereid had, maar Sjaan was hier niet over verrast.

? Kwam aan het licht dat Joost Hagestein de bijeenkomst niet had voorbereid? Y

28 b
Haar studenten kwamen erachter dat Joost Hagestein de bijeenkomst voorbereid had, maar Sjaan was hier niet over verrast.

? Kwam aan het licht dat Joost Hagestein de bijeenkomst niet had voorbereid? Y

28 c
Hij kwam erachter dat Joost Hagestein de bijeenkomst voorbereid had, maar Bert was hier niet over verrast.

? Kwam aan het licht dat Joost Hagestein de bijeenkomst niet had voorbereid? Y

28 d
Zij kwam erachter dat Joost Hagestein de bijeenkomst voorbereid had, maar Sjaan was hier niet over verrast.

? Kwam aan het licht dat Joost Hagestein de bijeenkomst niet had voorbereid? Y

29 a
Haar buren wisten niet dat Marja Locher het buurtfeest georganiseerd had, hoewel Wim wel een vaag vermoeden had.

? Had Marja Locher een vergadering georganiseerd? N

29 b
Zijn buren wisten niet dat Marja Locher het buurtfeest georganiseerd had, hoewel Wim wel een vaag vermoeden had.

? Had Marja Locher een vergadering georganiseerd? N

29 c
Zij wist niet dat Marja Locher het buurtfeest georganiseerd had, hoewel Pleun wel een vaag vermoeden had.

? Had Marja Locher een vergadering georganiseerd? N

29 d
Hij wist niet dat Marja Locher het buurtfeest georganiseerd had, hoewel Wim wel een vaag vermoeden had.

? Had Marja Locher een vergadering georganiseerd? N

30 a
Zijn collegas wisten zeker dat Huub Groothuis van fraude beschuldigd was, maar Greetje kon dit feit niet geloven.

? Werd Huub Groothuis van fraude beschuldigd? Y

30 b
Zijn collegas wisten zeker dat Huub Groothuis van fraude beschuldigd was, maar Greetje kon dit feit niet geloven.

? Werd Huub Groothuis van fraude beschuldigd? Y

30 c
Hij wist zeker dat Huub Groothuis van fraude beschuldigd was, maar Johan kon dit feit niet geloven.

? Werd Huub Groothuis van fraude beschuldigd? Y

30 d
Hij wist zeker dat Huub Groothuis van fraude beschuldigd was, maar Greetje kon dit feit niet geloven.

? Werd Huub Groothuis van fraude beschuldigd? Y

31 a
Haar ondergeschikten waren verbaasd dat Tjitske Draaijer een bonus zou krijgen, maar Willem had wel een vaag vermoeden.

? Moest Tjitske Draaijer salaris inleveren? N

31 b
Zijn ondergeschikten waren verbaasd dat Tjitske Draaijer een bonus zou krijgen, maar Willem had wel een vaag vermoeden.

? Moest Tjitske Draaijer salaris inleveren? N

31 c
Zij was verbaasd dat Tjitske Draaijer een bonus zou krijgen, maar Lara had wel een vaag vermoeden.

? Moest Tjitske Draaijer salaris inleveren? N

31 d
Hij was verbaasd dat Tjitske Draaijer een bonus zou krijgen, maar Willem had wel een vaag vermoeden.

? Moest Tjitske Draaijer salaris inleveren? N

32 a
Haar vrienden maakten duidelijk dat Noortje Helgers een kort geding aanspande, maar Vincent wist niet de exacte details.

? Is het waar dat Vincent niet de details wist? Y

32b

Haar vrienden maakten duidelijk dat Noortje Helgers een kort geding aanspande, maar Vincent wist niet de exacte details.

? Is het waar dat Vincent niet de details wist? Y

32 c
Zij maakte duidelijk dat Noortje Helgers een kort geding aanspande, maar Bea wist niet de exacte details.

? Is het waar dat Vera niet de details wist? Y

32 d
Hij maakte duidelijk dat Noortje Helgers een kort geding aanspande, maar Vincent wist niet de exacte details.

? Is het waar dat Vincent niet de details wist? Y

33 a
Zijn ploeggenoten merkten niet dat Casper Bakker een vervelende overtreding beging, maar Femke pretendeerde het gezien te hebben.

? Werd gemerkt dat Casper Schelvis een vervelende overtreding beging? N

33 b
Haar ploeggenoten merkten niet dat Casper Bakker een vervelende overtreding beging, maar Femke pretendeerde het gezien te hebben.

? Werd gemerkt dat Casper Schelvis een vervelende overtreding beging? N

33 c
Hij merkte niet dat Casper Bakker een vervelende overtreding beging, maar Jelle pretendeerde het gezien te hebben.

? Werd gemerkt dat Casper Schelvis een vervelende overtreding beging? N

33 d
Zij merkte niet dat Casper Bakker een vervelende overtreding beging, maar Femke pretendeer-de het gezien te hebben.

? Werd gemerkt dat Casper Schelvis een vervelende overtreding beging? N

34 a
Haar artsen maakten duidelijk dat Caro Petten geen medicatie nodig had, maar Meindert hield stiekem belangrijke informatie achter.

? Werd er belangrijke informatie achter gehouden? Y

34 b
Zijn artsen maakten duidelijk dat Caro Petten geen medicatie nodig had, maar Meindert hield stiekem belangrijke informatie achter.

? Werd er belangrijke informatie achter gehouden? Y

34 c
Zij maakte duidelijk dat Caro Petten geen medicatie nodig had, maar Merel hield stiekem belangrijke informatie achter.

? Werd er belangrijke informatie achter gehouden? Y

34 d
Hij maakte duidelijk dat Caro Petten geen medicatie nodig had, maar Meindert hield stiekem belangrijke informatie achter.

? Werd er belangrijke informatie achter gehouden? Y

35 a
Zijn reisgenoten maakten bekend dat Sander Hovink last van vliegangst had, dus Els nam direct de nodige maatregelen.

? Ontweek Els de situatie? N

35 b
Haar reisgenoten maakten bekend dat Sander Hovink last van vliegangst had, dus Els nam direct de nodige maatregelen.

? Ontweek Els de situatie? N

35 c
Hij maakte bekend dat Sander Hovink last van vliegangst had, dus Frank nam direct de nodige maatregelen.

? Ontweek Frank de situatie? N

35 d
Haar reisgenoten maakten bekend dat Sander Hovink last van vliegangst had, dus Els nam direct de nodige maatregelen.

? Ontweek Els de situatie? N

36 a
Haar opdrachtgevers kwamen erachter dat Wilma Kuipers een geheime verhouding had, dus Martijn had moeite met de situatie.

? Had Wilma een geheime verhouding? Y

36 b
Zijn opdrachtgevers kwamen erachter dat Wilma Kuipers een geheime verhouding had, dus Martijn had moeite met de situatie.

? Had Wilma een geheime verhouding? Y

36 c

Zij kwam erachter dat Wilma Kuipers een geheime verhouding had, dus Tirza had moeite met de situatie.

? Had Wilma een geheime verhouding? Y

36 d
Hij kwam erachter dat Wilma Kuipers een geheime verhouding had, dus Martijn had moeite met de situatie.

? Had Wilma een geheime verhouding? Y
